# The long horizon of animal study preregistration: Attitudes, barriers, and myths

**DOI:** 10.1371/journal.pbio.3003905

**Published:** 2026-07-29

**Authors:** Pasquale Pellegrini, Ulf Toelch

**Affiliations:** QUEST Center for Responsible Research, Berlin Institute of Health (BIH) at Charité – Universitätsmedizin Berlin, Berlin, Germany

## Abstract

Nearly half of Swiss animal researchers have never heard of preregistration. This Primer explores a survey published in PLOS Biology that maps their attitudes, barriers and misconceptionsm, and identifies what it would take to change minds.

## Teaser

Nearly half of Swiss animal researchers have never heard of preregistration. A survey published in *PLOS Biology* maps their attitudes, barriers, and misconceptions, and identifies what it would take to change minds.

Preregistration is the practice of publicly registering hypotheses, study designs, and analysis plans before data collection begins. This is not a new idea for clinical research, where clinical trials have been preregistered for over two decades [1]. However, this practice in preclinical research is still in early stages, with only 550 preregistered studies worldwide as of 2025 [2].

The rationale for preregistering a study is intuitive: if researchers commit to write down hypotheses and analysis plans before they collect data, subsequent papers will be less likely to contain results that are biased by questionable research practices. Amongst these are selective reporting (i.e., hiding negative data), p-hacking (all the tweaks done to ensure the highest statistical significance, such as outlier exclusion or *ad-hoc* modifications of the statistical test) and HARKing (hypothesizing after results are known). Such practices increase the chances of false positives and report exploratory data as more robust than it actually is: two major sources of poor reproducibility [3].

In a new survey in *PLOS Biology* led by Würbel and colleagues, the authors assessed the status of preregistration in Swiss animal research [4]. Of the 418 accredited study directors who completed the survey, nearly 40% had never even heard of preregistration and only one in 10 had ever done it. The findings arrive at a critical moment: funders and journals are increasingly moving toward preregistration requirements, yet the animal research community appears largely unprepared and resistant to such a shift.

Dedicated registries for animal studies exist (e.g., Preclinicaltrials.eu*,* Animalstudyregistry.org), yet uptake remains slow, and the reasons have, until now, been poorly characterized. Würbel and colleagues applied the theory of planned behavior (TPB), a framework from health psychology, to measure multiple psychosocial dimensions simultaneously: attitudes, subjective norms, perceived resources and knowledge, intentions, motivations, and perceived obstacles [4]. The survey also included open-ended questions on barriers, facilitators, and suggestions.

The results are consistent and striking. Across nearly all psychosocial constructs, participants scored on the negative side: unfavorable attitudes, social norms perceived as discouraging preregistration, limited perceived ability to preregister, and weak intentions and motivation. This contrasts sharply with survey data from psychology researchers in Germany, where 62% had prior preregistration experience and attitudes were generally positive [5]. The researchers from the current study who had previously preregistered their studies tended to have more favorable views toward preregistration. Years of experience in animal research were negatively associated with favorable views in preregistration, suggesting that a generational shift is ongoing, with younger scientists being more likely to preregister their research.

Interestingly, the perceived barriers to preregistration reported by survey participants were rooted, at least partly, in misconceptions. Most commonly reported were the bureaucratic burden, time costs, low flexibility, and fear of being scooped. For example, some researchers believed that preregistration is a rigid pre-plan that must be followed. However, there is always the possibility to add deviations later and report them transparently in the publication. As noted elsewhere, preregistration is a “plan, not a prison” [6]. Also, fear of scooping is unwarranted. On the contrary, preregistration protects against scooping by timestamping a research idea before results exist and allows for embargo (setting the preregistration as invisible to others). Targeted education addressing these misconceptions may therefore be among the most efficient interventions available at this stage.

Subtler patterns also emerged. Female researchers and those outside academia rated their preregistration knowledge lower than male or academic peers, a difference the authors interpret as potentially reflecting more accurate self-assessment by women than men. Researchers in general biology reported more favorable attitudes and fewer fears of being scooped than those in basic or applied biomedical research, possibly reflecting a less competitive disciplinary culture. These gradients suggest that promotion strategies and preregistration format will need to be tailored to the field, the hypothesis and the objective of the study.

A recurring theme in open-ended responses was frustration with Switzerland’s already demanding animal experimentation authorization process, with some participants viewing preregistration as redundant or as an additional bureaucratic layer. Although the animal permits and preregistration serve different purposes (i.e., ethics versus scientific rigor), there is indeed some content overlap between the two documents. This suggests that future methods of preregistration that are based on the animal permit itself could potentially reduce the workload on scientists.

Participants themselves pointed the way to better implement preregistration: flexible templates, confidentiality protections, dedicated support, and, above all, institutional and funder mandates. Nearly half indicated that policy requirements would influence their decision; almost a third said nothing would. In clinical research, the major turning point was when the editors of a top medical journal announced that preregistration was a condition for publication [1]. This points to a dual strategy: capacity building combined with regulatory enforcement. A possible starting point would be for regulators to require that data used to advance to clinical trials come from preregistered experiments.

It is worth noting that preregistration is not a guarantee against questionable research practices: preclinical researchers retain considerable degrees of freedom in outcome selection, analytical choice, and publication decision that a registered plan cannot fully constrain. This degree of freedom is arguably necessary to preserve flexibility in fields where research is inherently exploratory. For this, preregistration depth and granularity need to reflect the study character. Different templates that also allow for low effort and fast preregistration may serve as an entry point for researchers to this practice. Then, preregistration can move beyond a bureaucratic exercise and effectively serve its purpose to reliably improve pre-study planning and create a transparent record [2]. Whether such interventions will shift attitudes and behavior over time in samples beyond Switzerland will be worthwhile exploring. This will also enable researchers to investigate whether preregistration fulfills its intended goal of improving the credibility of animal studies.
